# Marathon Running and Sexual Libido in Adult Men: Exercise Training and Racing Effects

**Published:** 2022

**Authors:** Anthony C. Hackney, Gabriel H. Zieff, Amy R. Lane, Johna K. Register-Mihalik

**Affiliations:** 1 Department of Exercise & Sport Science, University of North Carolina, Chapel Hill, NC, USA; 2 Department of Nutrition, Gillings School of Global Public Health, University of North Carolina, Chapel Hill, NC, USA

**Keywords:** Running, Sexual desire, Sport, Testosterone, Low energy availability, RED-S

## Abstract

We examined whether endurance training for a standard marathon (42.2 km) had a greater influence on male libido than more generalized endurance exercise training. We surveyed adult men (>1000) who regularly engaged in endurance running to evaluate exercise training histories-patterns and libido characteristics. Our participants were primarily recruited from North America and Europe. Results indicate men conducting marathon training had lower libido scores (p<0.05; ~20%, d=0.44) than those not doing such specific training. Factors most related to libido were: 1) the number of years of training, and 2) the proportion of high-intensity effort conducted in training (inverse relationships); regardless of whether marathon training was performed or not. Our survey approach did not allow us to determine the cause of the reduced libido, but we speculate it could relate to: 1) chronic physical fatigue from high volumes of exercise training, 2) behavioral accommodations in energy expenditure, or else *‘Relative Energy Deficiency in Sport’* (RED-S) syndrome, and/or 3) endocrinological adaptations as a result of the exercise training (i.e., low testosterone). From a practical perspective, we recommend couples attempting conception should inform their healthcare providers of the male partner’s exercise habits concerning endurance running as this may be a factor relative to potential infertility.

## Introduction

Today more individuals are engaging in regular physical activity due to the ascribed health benefits. One of the most popular forms of physical activity is running, due to the simplicity of the activity and the lack of requirements for excessive equipment or specialized locations. In fact, in men distance running has been a leading health-fitness activity for decades^1^. Completing a marathon is a “bucket list” item for many such male runners. The recent occurrence of the 2-hour marathon being broken by Eliud Kipchoge of Kenya, and the return of mass running events such as the recent running of the 126^th^ Boston Marathon have fueled renewed interest in marathoning. Such interest is not limited to just elite-level runners either. For example, during the 10 years from 2008 to 2018, there were over 30,000 running races worldwide with nearly 20 million finisher results being reported^1^, with an increase in the number of participants observed every year. Notedly, while the sex distribution for these results is skewed towards men, women are an increasing number of participants, and as such in couples where each exercise they are/can be mutually supportive of one another in pursuing physical activity goals such as running a marathon.

Relative to men, our research group has reported that the level of sexual desire-arousal (i.e., libido) is reduced as they engaged in more chronic endurance exercise training of various types (e.g., running [road, track], triathlons, cross-country skiing…etc.) over longer periods of time (i.e., years in training)^2^. In light of the popularity of marathon running, we wondered whether the specific endurance training/racing for an event like it (high volume, long-distance running) had a greater influence on male libido than more general endurance exercise training /racing (i.e., distance running competitions less than the full marathon; e.g., 5 - 10 kilometer events or half-marathons). Understanding this overarching issue of libido is important as it is a factor in reproductive health and fertility in couples^3^.

## Materials and Methods

For this study, we conducted an online survey of male endurance athletes primarily from North America and Europe. The development and implementation of our survey were reviewed and approved by the University of North Carolina Institutional Review Board; and all participants gave informed consent (electronic). The survey questionnaire had been tested and evaluated to ensure its validity (psychometrics reported elsewhere^2^).

We received nearly 1400 completed survey responses during the one year the study was active (2016-17). Of these responses, 1080 met all study requirements to be appropriately analyzed (>18 years old, minimum of one year of regular endurance exercise training [≥3 times per week, ≥45 minutes per session], and a participant in competitive running events [road, trail and, or track]). All participants attested to being free of major health issues, especially known endocrine disorders, and not currently experiencing any musculoskeletal injury.

Of the eligible responses, 595 of the respondents indicated they were engaged in specific endurance training to complete a standard Olympic distance marathon (42.2 km). Libido and exercise training-competition components were assessed and scored based on prior published criteria^2^. Participants’ characteristics (range values) were as follows: age = 18 – 60 years, weight = 52 - 159 kg, height = 142 – 229 cm, training = 5 – 10+ years, training sessions = 3 – 10+ times per week, training time = 3 – 10+ hours per week (N.B., the proportion of training time [hours] at low, moderate and high-intensity levels were also assessed based upon the individuals rating of perceived exertion [see noted references for details])^2,4^.

Statistically, Chi-square, ANOVA, and logistic regression analyses were used to compare non-marathoners to marathoners, as well as within the marathoners (i.e., between those who had completed multiple such race events; categories being; 1, 2, 3, 4, +5 marathon races completed). Significance was set at a p<0.05 level. For significant findings, the effect size was determined using the Cohen *d* statistic. All statistical analysis was performed with SPSS software, version 21.

## Results

Men who were conducting marathon-specific training had lower libido scores (p<0.05; ~20%, *d*=0.44) than those who were not engaged in marathon training. Furthermore, as the number of marathons completed increased in these specific respondents, libido scores were reduced further, but not substantially (1 completed vs. +5 completed marathons resulted in approximately a further 12% greater reduction [p<0.10>0.05], *d*=0.15). Interestingly, in the overall sample (adjusted for age), the factors most associated with the libido score were the number of years in training (duration of exposure), and the proportion of high-intensity effort (low vs. moderate vs high exertion) at which that training was performed (displaying an inverse relationship for each factor), regardless of whether specific marathon training was being performed, or the number of marathons completed.

## Discussion

Our findings support that performing endurance exercise training (e.g., running) to complete a marathon was associated with reduced libido. However, years of training and the proportion of high-intensity training were the more critical overall determinates of the men’s libido score. That is, as men had greater exposure (time in years) to chronic endurance training, and the more of that training was done at a high intensity, the lower their libido score became. These specific findings support, to an extent, our earlier examination of this issue; but our prior work did not factor-in specifically the influence of the training/racing associated with a marathon (high volume, long-distance running) on libido reduction^2^.

Our survey methodology did not allow for causality assessment of the reduced libido finding in our runners, but others have attributed such occurrence to a chronic physical fatigue from high volumes of exercise training^5^, behavioral accommodations in energy expenditure (energetic “trade-offs” via the evolutionary biology perspective of the Life History Theory)^6^, and/or endocrinological adaptations as a result of the exercise training. Relative to the latter point, there is evidence in endurance-trained males there is a chronically reduced level of testosterone^7^. Specifically, testosterone values have been reported to be 10 – 40% lower than expected to be found in age-matched clinical reference men^7^.

Some researchers have speculated that related physiological aspects of the reduced libido, such as reduced morning erections, are linked to the state of Relative Energy Deficiency in Sport (RED-S) syndrome existing in athletic men and women^8^. We did observe a tendency for reports of reduced penal erection frequency (part of survey questions) as libido scores became reduced (within the entire sample), hence our outcomes could be related to RED-S development. Regrettably, assessment of RED-S is dependent on the measurement of energy availability in an individual (i.e., low energy availability precipitates RED-S)^9^, which is difficult to assess accurately in a laboratory yet alone in an online survey methodology^10^. Nonetheless we feel our data support that training athletes should be cognizant of their sexual drive as it may be an indicator of their energy status and a harbinger of RED-S development. Most certainly further research is needed to pursue this point on male reproduction function and exercise training^8,9^.

We acknowledge there are limitations to our study. Specifically, surveys tend to have sample participants of ‘convenience’ (i.e., a sample drawn from that part of the population that is close at hand and willing to participate), and as such limited in generalizability; although, the online nature of our survey broaden our sampling pool. Additionally, when asking questions concerning an individual’s ‘intimate life’ participants are known to reduce aspects of their truthfulness in responses^11^. Finally, we recognize while we had statistically significant outcomes, the effects sizes observed (Cohen *d*) were in the small to medium range^12^.

From a practical perspective, regardless of the mechanism for the reduced libido, our findings do suggest that reproductive health clinicians working with heterosexual couples attempting to conceive should make certain to question the exercise habits of male partners, especially with regard to endurance running for exercise training whether it is for a goal of completing a marathon or not. Likewise, we recommend the medical support team of competitive male endurance athletes readily inquire about the athlete’s libido status as part of their regular physical examinations, and to that end, endorse the use of questionnaires to assess such characteristics (e.g., LEAM-Q^8^ or Aging Male Symptoms^13^). This recommendation seems especially pertinent for those male athletes at high risk for low energy availability and RED-S development (see Mountjoy et al.)^9^.
